# Italian screening protocol and genotypes characterization for HCV elimination (2022–2023) in Ferrara’s province: a real-world study

**DOI:** 10.1038/s41598-025-92654-w

**Published:** 2025-03-08

**Authors:** Nicolò Landini, Chiara Chiericati, Michela Boni, Loredana Simone, Massimo Trombini, Chiara Zanforlin, Caterina Palmonari

**Affiliations:** 1 AUSL – U.O.S.D. Oncology Screenings, Epidemiology and Health Promotion Programs, Local Health Agency of Ferrara, Corso Giovecca 203, 44121 Ferrara, Italy; 2 Chemical-Clinical Analysis Laboratory, S. Anna University Hospital, Via Aldo Moro 8, 44124 Ferrara, Italy; 3 Department of Gastroenterology and Gastrointestinal Endoscopy, S. Anna University Hospital, Via Aldo Moro 8, 44124 Ferrara, Italy; 4 AUSL – Addiction Service, Local Health Agency of Romagna, Via Publio Ovidio Nasone 13, 47923 Rimini, Italy

**Keywords:** Hepatitis C, Population screening, Viral genotypes, Polymerase chain reaction, Prevention, Health system, Epidemiology, Disease prevention, Virology, Hepatitis C virus, Policy and public health in microbiology, Gastrointestinal diseases, Liver diseases, Hepatitis, Viral hepatitis, Hepatitis C, Hepatology, Hepatitis, Viral hepatitis, Hepatitis C, Health policy, Patient education, Public health, Population screening, Diagnosis, Laboratory techniques and procedures, Pathology, Medical research, Epidemiology

## Abstract

Hepatitis C virus (HCV) is a worldwide health hazard, and in chronic form (nowadays affecting 50 million people – World Health Organization data) can be lethal. To forestall it, preventive screening is a mandatory approach. Since 2021, Italy conducts a national-wide screening program to eliminate the virus from its population. The team perfected an innovative method throughout the period between 2022 and 2023, to answer that medical necessity and map HCV genotypes. The medical protocol has introduced a dedicated double invitation model for the adherents, with consequential pre-prepared medical consumables allocated. The population was divided in three separate groups: born between 1969 and 1989, addiction services, and prison. Two screening levels were carried out: anti-HCV antibodies (indirect chemiluminescence immunoassay) and quantitative HCV RNA reverse transcription. 51,283 adherents were registered: 447 resulted positive to the first screening round, and 88 to the second (393 and 77 patients respectively from population born between 1969 and 1989). The medical protocol introduced allowed to substantially increase patients and medical staff compliance to HCV screening (adherence: 51.83%, the highest in Italy). HCV genotypes’ distribution was mapped by patients’ age, biological sex and origin over five groups comprehending subtypes 1a(35.06% of the positive population), 1b(27.27%), 2a/2c(10.39%), 3a(22.08%), 4a/4c/4d(5.19%).

**Supplementary Information:**

The online version contains supplementary material available at 10.1038/s41598-025-92654-w.

## Introduction

### Hepatitis C virus

Hepatitis C virus (HCV), a bloodborne virus, is the causing factor of the homonymous infection. It can cause lifelong serious illnesses, in acute and chronic states. In 2024, the World Health Organization (WHO) estimated that, worldwide, approximately 50 million people had chronic HCV infection (of which 3.2 million adolescents and children), and approximately 242,000 people died from hepatitis C due to the complications previously cited in 2022. About 1.0 million new infections occur each year^1^.

Just in 2020, European Union (EU) reported 13,914 cases of hepatitis C, between acute (837), chronic (4,917), unknown (7,415) statuses of the illness, and 745 which could not be classified due to incompatibilities in data format^2^. In Italy in particular, HCV prevalence in the population is 0.66% with 95% Confidence Interval: 0.66–0.67, as per Kondili et al. (2021)^3^.

HCV infections mostly occur due to the following causes, ordered here by chronic condition occurrence^1–5^: drugs injection (syringes re-use), nosocomial (hospitals, nursing homes, psychiatric and dental institutions, unsafe health-care environment), non-occupational injuries (unsafe injection practices outside healthcare setting, cosmetic surgery, bites, tattoos, piercings), blood and blood products, unsafe sexual practices (any type leading to exposure to blood), needle stick and other occupational exposure (comprehends unscreened blood transfusions and other staff-dependent causes), perinatal and mother-to-child transmission, household, hemodialysis.

HCV infection main chronic late-stage pathology is liver cirrhosis^6^, in which healthy liver tissue is gradually replaced with scar tissue. It is a progressive condition, which worsens with the scar tissue outgrowing. The human body initially adjusts to compensate the reduced liver function (compensated cirrhosis), thus not alarming the patient with notable symptoms. Eventually, though, as the liver functions decline further, harmful symptoms will eventually occur (decompensated cirrhosis), up to eventual liver failure and death if not treated. Another complication that may spawn from cirrhotic liver is the HepatoCellular Carcinoma (HCC)^7^, a type of liver malignant neoplasia.

### Direct-acting antiviral

HCV infection can be cured in more than 95% of the patients with direct-acting antiviral medicines (DAAs) with 8–16 weeks therapeutic cycles^8^.

Historically, interferon proteins used to be the first drugs of choice to help the organism to heal from chronic hepatitis C. Then, the treatment evolved first by coupling interferons with Ribarivin, and then with the introduction of protease inhibitors Telaprevir (TVR) and Boceprevir in 2011 (the latter two being the first DAAs). Afterwards, interferons treatments were mostly abandoned for first and second generation DAAs, up until present days in which combinations and C-range drugs are used to cure the pathology. As per ministerial regulations^9^, Sofosbuvir/Velpatasvir^8–10^, Glecaprevir/Pibrentasvir^8,9,11^ are the DAAs used from S. Anna University Hospital. They have pangenotypic properties, and act directly by blocking virus replication process.

### New and foreign HCV strains

As the majority of known viral agents, HCV presents itself in various genotypes and subtypes. From 2022 mapping, the known HCV genotypes are eight, with 86 different subtypes^12^.

As per literature^12,13^, it has been observed that some genotypes are predominant dependently the geographical area. Said distribution is summarized in the following list^12,14^: North America: 1a, Europe: 1b, Italy: 1b, Far East: 2, Central Asia: 3, Middle East, Africa: 4, Southern Africa: 5, Southeast Asia: 6.

For the Italian case, studies on HCV genotypes showed in 2019 how genotypes from 1 to 4 are more common in the national territory, with prevalence on the variants 1b and 2c, followed by 1a, 3a and 4a/d ^14,16^.

### Criticalities

Despite the effectiveness of DAAs usage from physicians on patients to treat HCV infections, access and adherence from the population to diagnosis and treatment in order to counter HCV infection were still low. In fact, the public opinion on the matter is heavily, negatively biased, flawed from different factors. There is a systematic factor, due to the high infectivity of the disease, and the lack of proper funding from the NHS to provide for the expenses required. There is as well an organizational factor, due to lacks of proper preparation between nurses and physicians on the matter when referred to advances in screening and treatment, and consequential use of outdated medical protocols. Finally, also a social factor exists, due to the erroneous or flawed public sensibility and knowledge on the concept of HCV infection (sexual, environmental and recreational dependent transmissions, and adoption of not-knowing culture by avoiding screening, due to the erroneous belief in the impossibility of healing from the disease once contracted).

This results in indifference and even a taboo approach from the public on the matter, even in the most advanced countries. These criticalities, together with the additional negative factor that there is currently no vaccine against hepatitis C, play the role of major obstacles in the battle to eradicate HCV.

Before the recent national HCV eradication program instauration in 2021, HCV screening was not active at all in Italy (e.g.: in Ferrara’s Province, only spontaneous applicant tested themselves, after request at their primary care physician), and was free-of-charge only for few categories (e.g.: pregnant women).

For these reasons, three actions have been taken to enhance early HCV diagnosis and intervention, before its degeneration into harmful or lethal illnesses^17,18^. First of all, steady and reliable screening protocols were established from the NHS; secondly, a proper information campaign was conducted on risks and healing chances, aimed to sensibilize and responsibilize the public; finally, new training activities was held for the involved medical staff, in order to properly address the infection control.

### Target of the study

Given the medical problem represented from HCV infections diffusion, the aims of this work are to verify the effectiveness of the medical policies introduced and adopted from the Ferrara’s AUSL unit, by observation of the collected HCV screening compliance data, and to discern HCV genotype and subtypes prevalences in Ferrara’s province.

In order to eliminate HCV, Italy started a national experimental free-of-charge screening program for the most susceptible population age range (1969–1989, considered the most vulnerable to HCV infection^19^) in 2021 ^20^, coupling it with already existing screening programs on SerD (Addiction Services) and prison populations, likewise vulnerable to the infection. The range of birth between 1969 and 1989 was chosen following epidemiologic, economical and ethical motives derived from previous studies ^19^. Some of these motives are, but are not limited to: track the spread of HCV in the segment of the population in which it is verified its highest incidence, and in particular, quantify the not yet diagnosed infections that could remain asymptomatic for a long period of time; abide to the economic feasibility, defined at a national level, for organized screening coverage in the most vulnerable segments of the population; and track down the infections that occurred the 20 years period of time preceding the first isolation of the HCV virus, diagnosed in 1989, which prompted the start to the possibility of undergoing voluntary screening to detect this specific type of hepatitis.

Individuals belonging to the older generation (born between 1948 and 1968) and the younger generation (born from 1990 onwards) who are not actively invited in the screening program have the option of undergoing HCV testing, following the request or advice from their general practitioner.

The results expected overall from this screening are the identification of hepatitis C infections still asymptomatic and unknown (submerged); to pre-emptively treat patients to avoid advanced liver diseases and extrahepatic manifestations; to stop the virus circulation, thus preventing new infections; to guarantee on an experimental basis to the entire population registered in the health register, including Foreigners temporarily present (STP), the active offer of the HCV REFLEX until 31 December 2024; and to ensure the annual collection of activity data subject to regional and national reporting.

## Methods

### Ethics

All research was conducted in accordance with both the Declarations of Helsinki and Istanbul.

The work subject is an Italian national-wide screening program, not a clinical trial, therefore not only the connection between patients and personal data cannot be traced and no patient consent was required, but said data are part of the evolution of the *Information Obligation* for the *NSIS* (*New sanitary informative system*) and *SIAD flux* (*Homecare Assistance*) in the Healthcare Environment required by the relevant national bodies (Region, Ministries, Corte dei Conti) for statistical analysis. Regulations of said protocol are fetchable in the correlated Decree-Laws, available indefinitely at: DECRETO 14/05/2021, “Esecuzione dello screening nazionale per l’eliminazione del virus dell’HCV.” (21A04075) (GU Serie Generale n.162 del 08-07-2021). https://www.gazzettaufficiale.it/eli/id/2021/07/08/21A04075/sg/. (Accessed November 13, 2024); D.P.C.M. May 17, 1984 and s.m. https://www.salute.gov.it/imgs/C_17_pubblicazioni_1916_allegato.pdf (Accessed November 13, 2024); D.M. December 5, 2006. https://www.trovanorme.salute.gov.it/norme/dettaglioAtto.spring?id=24079 (Accessed July 1, 2024); DECRETO 7 dicembre 2016, n. 262. https://www.gazzettaufficiale.it/eli/id/2017/02/08/17G00016/sg (Accessed November 13, 2024).

### Protocol and enlisting

The total population was divided in three TARGET groups:

1. Population in the critical age range, spanning births from 1969 to 1989, with examination cohorts: 93,755 people in 2022, and 97,622 people in 2022–2023 (TARGET 1).
2. SerD, total population screening (TARGET 2).
3. Prison, total population, for which medical screenings are mandatory (TARGET 3).

The three TARGET populations are mutually exclusive. Each individual is associated with a single TARGET population group, contingent on their legal and health status.

In particular for TARGET 1 population, total compliance is divided into two categories, Informed and Spontaneous. Informed adherence, labelled as “Adherence”, refers to the TARGET 1 population segment that underwent HCV screening after receiving and complying with the standard invitation, in accordance with the national protocol. Spontaneous adherence, labelled as “Spontaneous”, refers to the TARGET 1 population segment that underwent HCV screening while scheduled for other blood tests, due to the activation of Ferrara’s customized double-invitation protocol (by sending an SMS the day prior to a routine blood sampling). So, while “Adherence” data (from informed adherents) refer to patients invited normally following the national protocol, “Spontaneous” data (from spontaneous adherents) refers to patients that, together with the active national invitation from the Personal Health Record, the day before any routine blood sampling were sent a dedicated SMS message, resulting therefore in a double-invitation.

The SMS embedded a web-link asking for the participation willingness to the HCV screening, which under acceptance opens the complete screening medical policy, explaining the screening program in all its steps, and prints the HCV blood sample’s tags for the secretary, so that the next day the vials are labelled for the sampling.

### Laboratory testing in HCV screening

Ferrara’s province started its free-of-charge population screening protocol on 20/12/2021^17–20^. The sampling strategies on venous blood samples utilized as HCV screening methods [21–24] in this work are the following:

1st level: HCV serologic testing – Antibodies (Ab) anti-HCV.

2nd level: HCV RNA testing – Quantitative HCV RNA polymerase chain reaction (PCR). Consequentially to step 2, HCV genotype testing on the same samples previously tested is carried out.

Starting from 2022, population compliance preliminary data started to be registered regionally^25^. With this study, the full HCV screening results on said population (years 2022–2023) have been produced.

All analyses were performed at the Clinical Pathology Unit, Ferrara University Hospital (UOC Patologia Clinica, Azienda Ospedaliero-Universitaria di Ferrara).

Two different levels of laboratory testing on venous blood were planned on the basis of dedicated algorithms (REFLEX testing), with the 2nd level contextually carried out on all the 1st level positive results. The 1st level was carried out to detect anti-HCV antibodies in serum. The serologic assay used for qualitative determination of specific IgG to HCV was an indirect chemiluminescence immunoassay (CLIA)^26^. The 2nd level was carried out on HCV-antibodies positive samples to assess active Vs resolved infections. A Quantitative HCV RNA Reverse Transcription Polymerase Chain Reaction (RT-qPCR) was performed to detect and quantitate hepatitis C virus RNA in plasma^27^. It was then performed a Line ImmunoAssay (LIA)^28^, designed to identify HCV genotypes and subtypes, in all specimens with detectable viral load. In case of undetermined result or very low level of viremia, the genotype was defined by Next-Generation Sequencing Technologies (NGS)^29^ at Microbiology Unit, AUSL Romagna (UO Microbiologia, Laboratorio Unico Pievesestina, Forlì-Cesena).

Blood samples were collected aseptically by venipuncture, using two different collection tubes for serum and plasma. For all serologic and molecular tests, commercially available kits were used.

### Data analysis

The values in Table 1 are calculated as follows:

$$\% ~adherence~to~HCV~1{\text{st}}~level~analysis~=~\frac{{Total~screened~\left( B \right)}}{{Invited~\left( A \right)}}$$

$$\% ~positive~to~1{\text{st}}\,level~HCV~screening~=~\frac{{Positive~to~1{\text{st}}~level~HCV~screening~\left( C \right)}}{{Total~screened~\left( B \right)}}$$

$$\% ~positive~to~2{\text{nd}}~level~HCV~screening~=~\frac{{Positive~to~2{\text{nd}}~level~HCV~screening~\left( D \right)}}{{Total~screened~\left( B \right)}}$$

$$\% ~positive~to~2{\text{nd}}~level~HCV~screening~over~1{\text{st}}~level~positives~=~\frac{{Positive~to~2{\text{nd}}~level~HCV~screening~\left( D \right)}}{{Positive~to~1{\text{st}}~level~HCV~screening~\left( C \right)}}$$

The screening indicators in Table 2 are calculated as follows:

$$Extension=\frac{{Invited~to~the~screening~test~\left( F \right)}}{{Eligible~population~\left( E \right)}}$$

$$Compliance=\frac{{Taking~the~screening~test~\left( G \right)}}{{Invited~to~the~screening~test~\left( F \right)}}$$

$$Coverage=\frac{{Taking~the~screening~test~\left( G \right)}}{{Eligible~population~\left( E \right)}}$$

$$Positive~Ab~research~test=\frac{{Positive~to~the~screening~test~\left( H \right)}}{{Taking~the~screening~test~\left( G \right)}}$$

$$Adherence~to~the~confirmatory~test=\frac{{Taking~the~confirmatory~test~\left( I \right)}}{{Positive~to~the~screening~test~\left( H \right)}}$$

$$Confirmatory~test~positivity=\frac{{Positive~in~the~confirmatory~test~\left( J \right)}}{{Taking~the~confirmatory~test~\left( I \right)}}$$

$$Detection~Rate=\frac{{Positive~in~the~confirmatory~test~\left( J \right)}}{{Taking~the~screening~test~\left( G \right)}} \cdot ~1,000$$

$$Percentage~of~subjects~with~active~infection~started~on~treatment=\frac{{Starting~therapeutic~treatment~\left( K \right)}}{{Positive~in~the~confirmatory~test~\left( J \right)}}$$

All percentages are calculated between total and partial populations depending on single genotypes, origin and biological sex. Due to software approximations, percentage values sums may not always result equal to 100%, but to 99.99–99.98%.

## Results

Results from the whole screening protocol on TARGET 1 population are summarized in Table 1, divided into two different time frames (one year for the sub-table labelled as “2022” and two years for the sub-table labelled as “2022–2023”) in order to facilitate the visualization of the behavior of the population in terms of compliance with the protocol over time. Table 1 presents for both time frames the number citizens, per year of birth and sex, which have been invited, that took the screening test under “Adherents” or “Spontaneous” protocol as described in the “Protocol and Enlisting” subchapter, and the patients found positive to the 1st and 2nd level of screening. Consequently from these data, percentage indexes on adherence and positivity rates are listed, calculated as described in the pertinent “Data Analysis” subchapter. Percentage of adherence to 2nd level HCV screening was substantially 100% between all ages and biological sexes, so to avoid redundancy it was not shown in the table.

**Table 1:** HCV screening adherents (Ferrara’s Province, born between 1969–1989).

Year of birth	Invited (A)	Total screened (B)	Adherence	Spontaneous	Positive to 1st level HCV screening (C)	Positive to 2nd level HCV screening (D)	% adherence to HCV 1st level analysis (B/A)	% positive to 1st level HCV screening (C/B)	% positive to 2nd level HCV screening (D/B)	% positive to 2nd level HCV screening over 1st level positive (D/C)
**YEAR 2022**										
1969	5,969	2,024	1,323	701	17	4	33.91%	0.84%	0.20%	23.53%
1970	5,792	2,338	1,346	992	30	3	40.37%	1.28%	0.13%	10.00%
1971	5,722	2,273	1,321	952	34	3	39.72%	1.50%	0.13%	8.82%
1972	5,872	2,315	1,372	943	20	1	39.42%	0.86%	0.04%	5.00%
1973	5,673	2,088	1,249	839	15	4	36.81%	0.72%	0.19%	26.67%
1974	5,782	2,207	1,268	939	10	1	38.17%	0.45%	0.05%	10.00%
1975	5,368	1,979	1,201	778	20	3	36.87%	1.01%	0.15%	15.00%
1976	5,182	1,948	1,180	768	16	4	37.59%	0.82%	0.21%	25.00%
1977	4,889	1,781	1,103	678	15	6	36.43%	0.84%	0.34%	40.00%
1978	4,559	1,660	993	667	10	2	36.41%	0.60%	0.12%	20.00%
1979	4,252	1,592	935	657	10	2	37.44%	0.63%	0.13%	20.00%
1980	3,876	1,404	890	514	10	1	36.22%	0.71%	0.07%	10.00%
1981	3,747	1,410	837	573	4	0	37.63%	0.28%	0.00%	0.00%
1982	3,661	1,366	811	555	5	1	37.31%	0.37%	0.07%	20.00%
1983	3,644	1,367	799	568	3	0	37.51%	0.22%	0.00%	0.00%
1984	3,443	1,312	779	533	7	2	38.11%	0.53%	0.15%	28.57%
1985	3,405	1,218	722	496	7	1	35.77%	0.57%	0.08%	14.29%
1986	3,306	1,225	720	505	8	0	37.05%	0.65%	0.00%	0.00%
1987	3,114	1,163	663	500	3	1	37.35%	0.26%	0.09%	33.33%
1988	3,288	1,234	731	503	2	1	37.53%	0.16%	0.08%	50.00%
1989	3,211	1,094	637	457	1	0	34.07%	0.09%	0.00%	0.00%
Female	46,619	21,961	12,767	9,194	121	18	47.11%	0.55%	0.08%	14.88%
Male	47,136	13,037	8,113	4,924	126	22	27.66%	0.97%	0.17%	17.46%
Both sexes	93,755	34,998	20,880	14,118	247	40	37.33%	0.71%	0.11%	16.19%
**YEARS ** **2022–2023**										
1969	6,098	2,970	2,170	800	28	7	48.70%	0.94%	0.24%	25.00%
1970	5,927	3,216	2,147	1,069	46	7	54.26%	1.43%	0.22%	15.22%
1971	5,856	3,257	2,230	1,027	41	5	55.62%	1.26%	0.15%	12.20%
1972	6,024	3,314	2,284	1,030	35	6	55.01%	1.06%	0.18%	17.14%
1973	5,837	3,068	2,148	920	26	7	52.56%	0.85%	0.23%	26.92%
1974	5,945	3,097	2,075	1,022	23	3	52.09%	0.74%	0.10%	13.04%
1975	5,515	2,797	1,942	855	27	3	50.72%	0.97%	0.11%	11.11%
1976	5,361	2,780	1,927	853	29	11	51.86%	1.04%	0.40%	37.93%
1977	5,064	2,550	1,794	756	20	6	50.36%	0.78%	0.24%	30.00%
1978	4,711	2,451	1,706	745	17	4	52.03%	0.69%	0.16%	23.53%
1979	4,424	2,263	1,542	721	19	5	51.15%	0.84%	0.22%	26.32%
1980	4,067	2,018	1,428	590	15	2	49.62%	0.74%	0.10%	13.33%
1981	3,914	2,066	1,413	653	11	2	52.78%	0.53%	0.10%	18.18%
1982	3,849	1,948	1,304	644	11	2	50.61%	0.56%	0.10%	18.18%
1983	3,845	1,995	1,327	668	4	0	51.89%	0.20%	0.00%	0.00%
1984	3,657	1,930	1,292	638	12	4	52.78%	0.62%	0.21%	33.33%
1985	3,626	1,849	1,240	609	9	1	50.99%	0.49%	0.05%	11.11%
1986	3,512	1,874	1,250	624	9	0	53.36%	0.48%	0.00%	0.00%
1987	3,413	1,800	1,179	621	6	1	52.74%	0.33%	0.06%	16.67%
1988	3,539	1,887	1,266	621	4	1	53.32%	0.21%	0.05%	25.00%
1989	3,438	1,638	1,073	565	1	0	47.64%	0.06%	0.00%	0.00%
Female	48,412	31,029	20,652	10,377	172	27	64.09%	0.55%	0.09%	15.70%
Male	49,210	19,739	14,085	5,654	221	50	40.11%	1.12%	0.25%	22.62%
Both sexes	97,622	50,768	34,737	16,031	393	77	52.00%	0.77%	0.15%	19.59%

Ferrara’s province HCV screening (years 2022 and 2022–2023) data sheet per years of birth on TARGET 1 group. “Adherence” = TARGET 1 population segment which underwent HCV screening after receiving and complying to the invite as per national protocol; “Spontaneous” = TARGET 1 population segment which underwent HCV screening voluntarily before receiving the invite, autonomously, or before the national screening started.

In Table 2, Ferrara’s province total and single TARGET populations HCV screening and positivity data are summarized for both time frames. The total number of citizens involved in the screening categorized for singular TARGET populations and their overall sum (“Total” column), are here presented. The citizens are sorted by into eligible population, invited population, adherents to the screening, positives to the 1st level, adherents to the 2nd level, positive to the 2nd level, sent to specialized centers, undergoing the specialist visit and taking the therapeutic treatment, each a subset of the previous group. Consequently from these data, screening indicators are listed, calculated as described in the pertinent “Data Analysis” subchapter.

**Table 2:** Ferrara’s Province total and single TARGET populations HCV screening and positivity.

	FERRARA (2022)				FERRARA (2022–2023)			
No. of subjects	Population born between 1969 and 1989 (TARGET 1)	SerD (TARGET 2)	Prison (TARGET 3)	Total	Population born between 1969 and 1989 (TARGET 1)	SerD (TARGET 2)	Prison (TARGET 3)	Total
Eligible population (E)	96,848	134	205	97,187	100,201	1,445	429	102,075
Invited to the screening test (F)	93,755	50	205	94,010	97,622	900	429	98,951
Taking the screening test (G)	34,998	17	205	35,220	50,768	105	410	51,283
Positive to the screening test (H)	247	1	17	265	393	13	41	447
Taking the confirmatory test (I)	247	0	17	264	391	10	41	442
Positive in the confirmatory test (active HCV infection identified) (J)	40	0	7	47	77	2	9	88
Sent to specialist treatment centres	38	0	7	45	77	0	9	86
Undergoing specialist visit	28	0	7	35	60	0	9	69
Starting therapeutic treatment (K)	28	0	7	35	60	0	9	69

Screening indicators								
Extension (F/E)	96.81%	37.31%	100.00%	96.73%	97.43%	62.28%	100.00%	96.94%
Compliance (G/F)	37.33%	34.00%	100.00%	37.46%	52.00%	11.67%	95.57%	51.83%
Coverage (G/E)	36.14%	12.69%	100.00%	36.24%	50.67%	7.27%	95.57%	50.24%
Positive Ab research test (H/G)	0.71%	5.88%	8.29%	0.75%	0.77%	12.38%	10.00%	0.87%
Adherence to confirmatory test (I/H)	100.00%	0.00%	100.00%	99.62%	99.49%	76.92%	100.00%	98.88%
Confirmatory test positivity (J/I)	16.19%	–	41.18%	17.80%	19.69%	20.00%	21.95%	19.91%
Detection Rate (J*1,000/G)	1.14	0.00	34.15	1.33	1.52	19.05	21.95	1.72
Percentage of subjects with active infection on treatment (K/J)	70.00%	–	100.00%	74.47%	77.92%	0.00%	100.00%	78.41%

Ferrara’s province HCV screening and positivity data (years 2022 and 2022–2023).

**Fig. 1:**
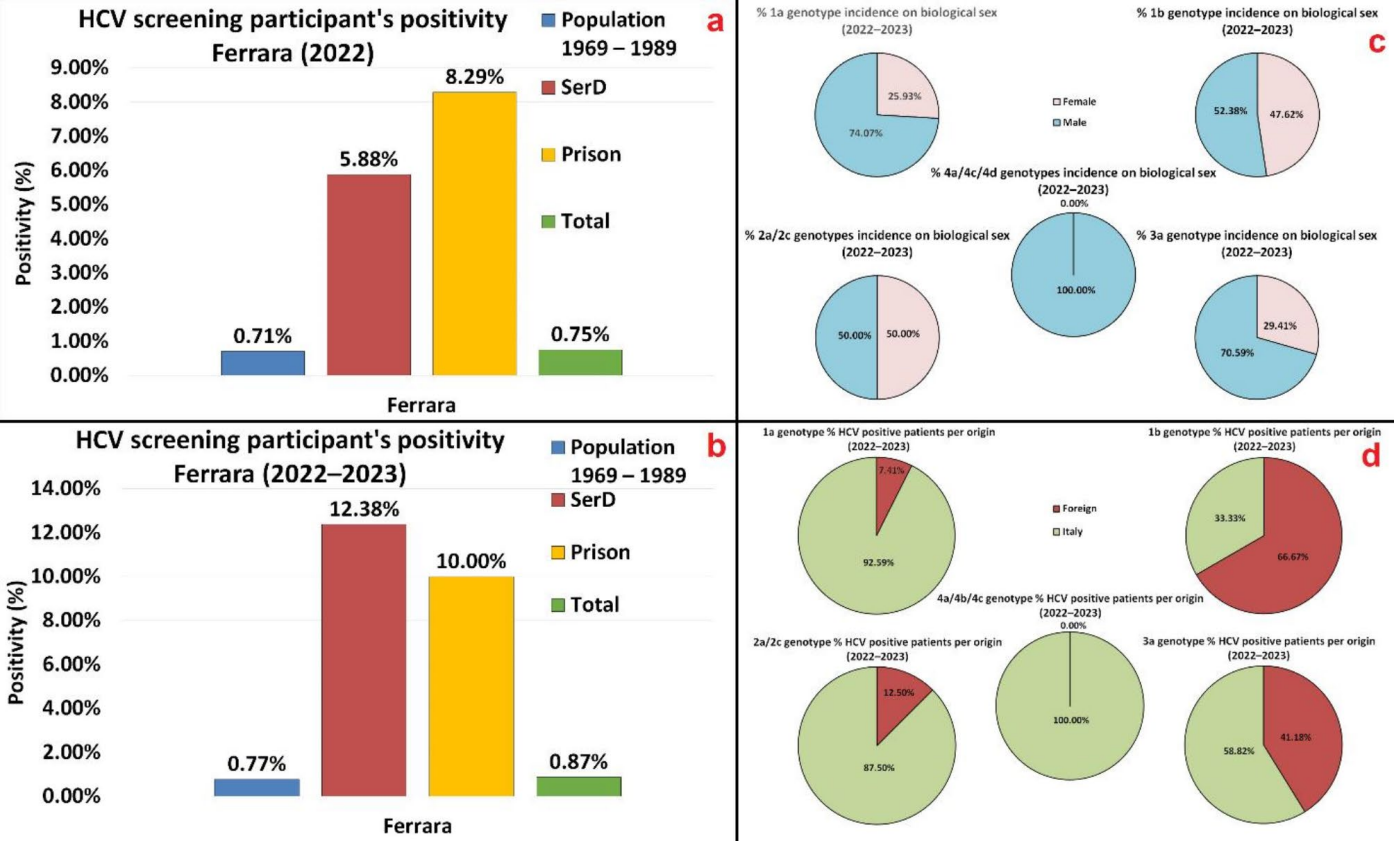
Ferrara’s province HCV screening participants’ data positivity percentage, divided by TARGET groups: (**a**) 2022. (**b**) 2022–2023. Genotype incidence, over the single genotype occurrence, in respect to: (**c**) Biological sex. (**d**) Origin.

Due to the most common HCV contagion route (drugs injection), populations undergoing SerD rehabilitation courses, or confined in prison, showed greater positivity percentage compared to the population average (see also Fig. 1).

**Fig. 2:**
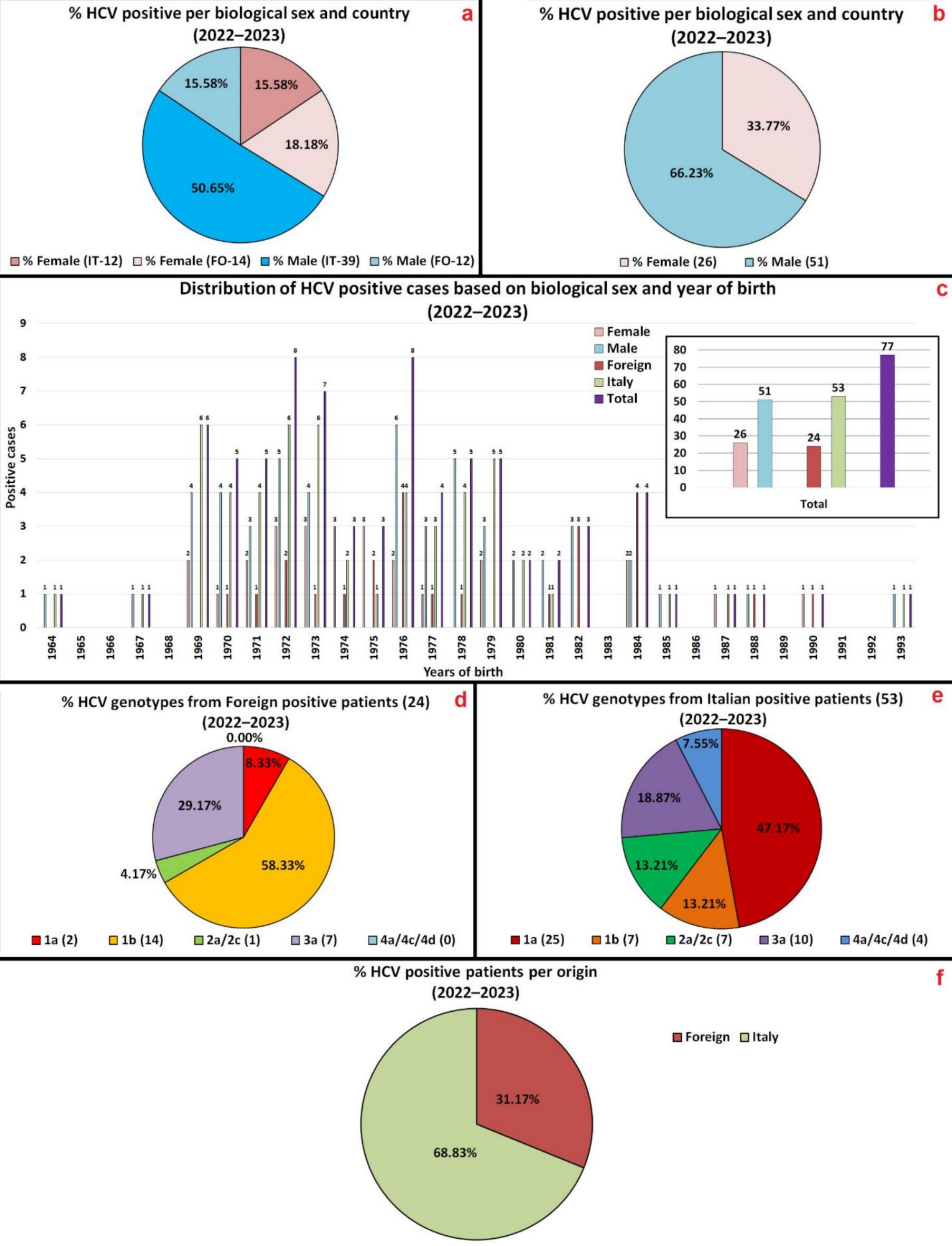
HCV screening participants’ positivity incidence and data (2022–2023) for Ferrara’s province: (**a**) By origin and biological sex. (**b**) By biological sex. (**c**) Number of positive cases, divided by year of birth and biological sex. (**d**) On Foreign positive patients. (**e**) On Italian positive patients. (**f**) On total positive patients.

**Fig. 3:**
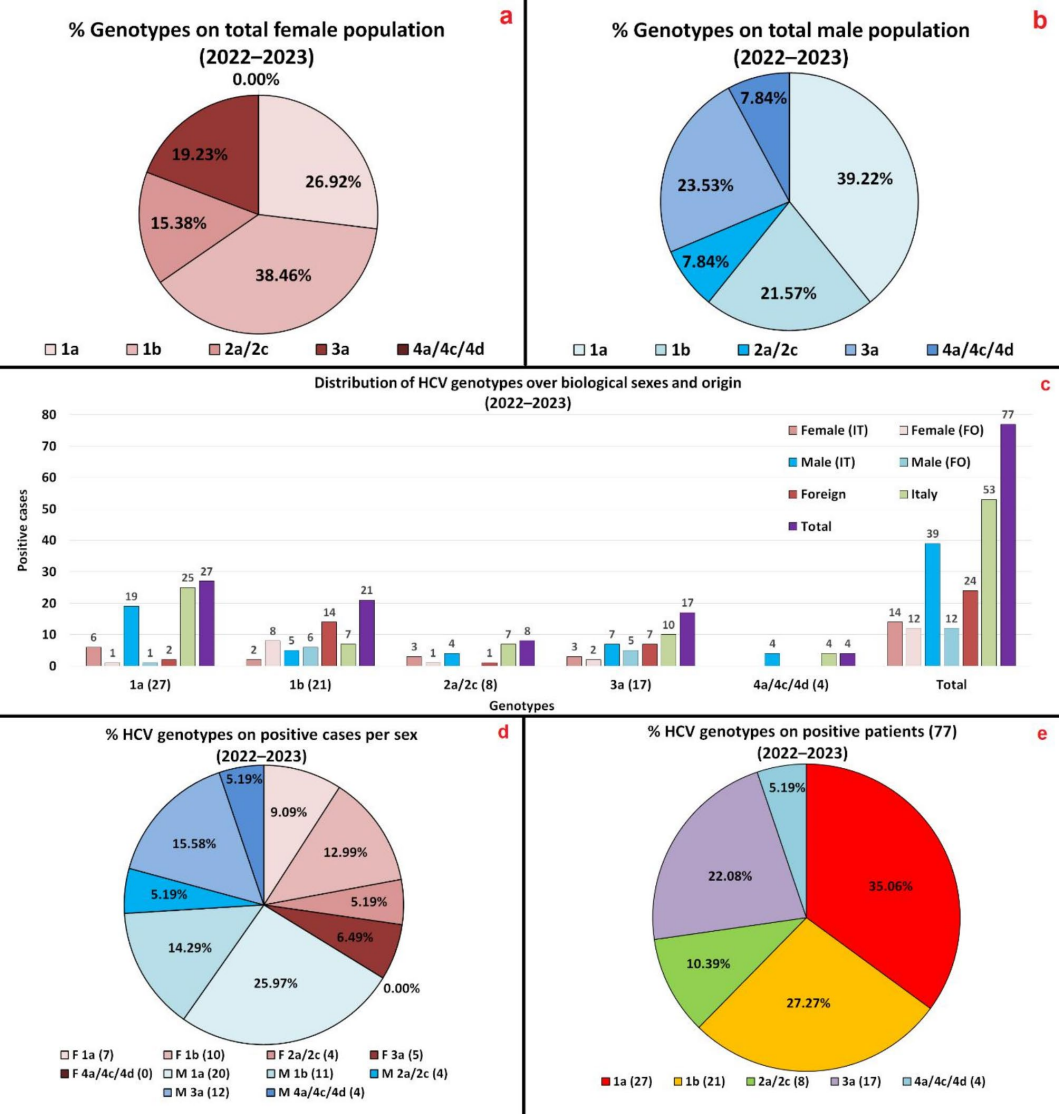
HCV genotype incidence data (2022–2023) for the Ferrara’s province: (**a**) On total female population. (**b**) On total male population. (**c**) Distribution on the positive population over biological sex and origin. (**d**) By genotype and biological sex. (**e**) By genotype.

In the following Figs. 1, 2 and 3 (referring to Tables S1–S5 in Supplementary Material), HCV genotypes prevalence data are shown. Once verified, the data were catalogued by geographical origin (Italy Vs Foreigners – STP, with the latter group including patients native to Pakistan, Ukraine, Moldova, Russia, Bulgaria, Cameroon, and Romania), year of birth and biological sex. The genotypes subtypes have been divided in five groups, following their occurrence between positive cases: 1a, 1b, 2a/2c, 3a, and 4a/4c/4d.

Figure 1 presents two histograms showing, in percentage, the screening participants positivity to the 1st level of HCV screening, sorted by TARGET groups and overall, referring respectively for the 2022 (a) and 2022–2023 (b) time frame. The same figure presents also two sets of pie charts, referred to TARGET 1 population, listing the various genotype incidence over their pertinent group occurrence abovementioned, in respect to the patient’s biological sex (c) and origin (d).

Figure 2, which refers to data from TARGET 1 population, presents three pie charts in which the percentages components on the total of the patients positive to the 1st level of screening can be visualized, sorted by biological sex together with the origin (a), and solely by biological sex (b), and origin (f). It presents also a histogram (c) in which the distribution of the patients positive to the 1st level of screening is sorted by year of birth (greater graphic), and by biological sex and origin (smaller graphic –the sum of the data divided per year). Finally it displays two more pie charts, in which the genotype group of the positive case to the 1st level of screening are sorted by origin, first for the Foreign population subgroup (d), and then for the Italian subgroup (e).

Figure 3 which refers to data from TARGET 1 population, presents four pie charts in which the genotype groups are sorted on biological sex for: the sole female subgroup (a); the sole male subgroup (b); the total population, in respect to both biological sexes (d); and on the total population without distinction on biological sexes (e). It presents also a histogram (c) in which the distribution of genotype groups on patients positive to the 1st level of screening is sorted by origin and biological sex.

**Fig. 4:**
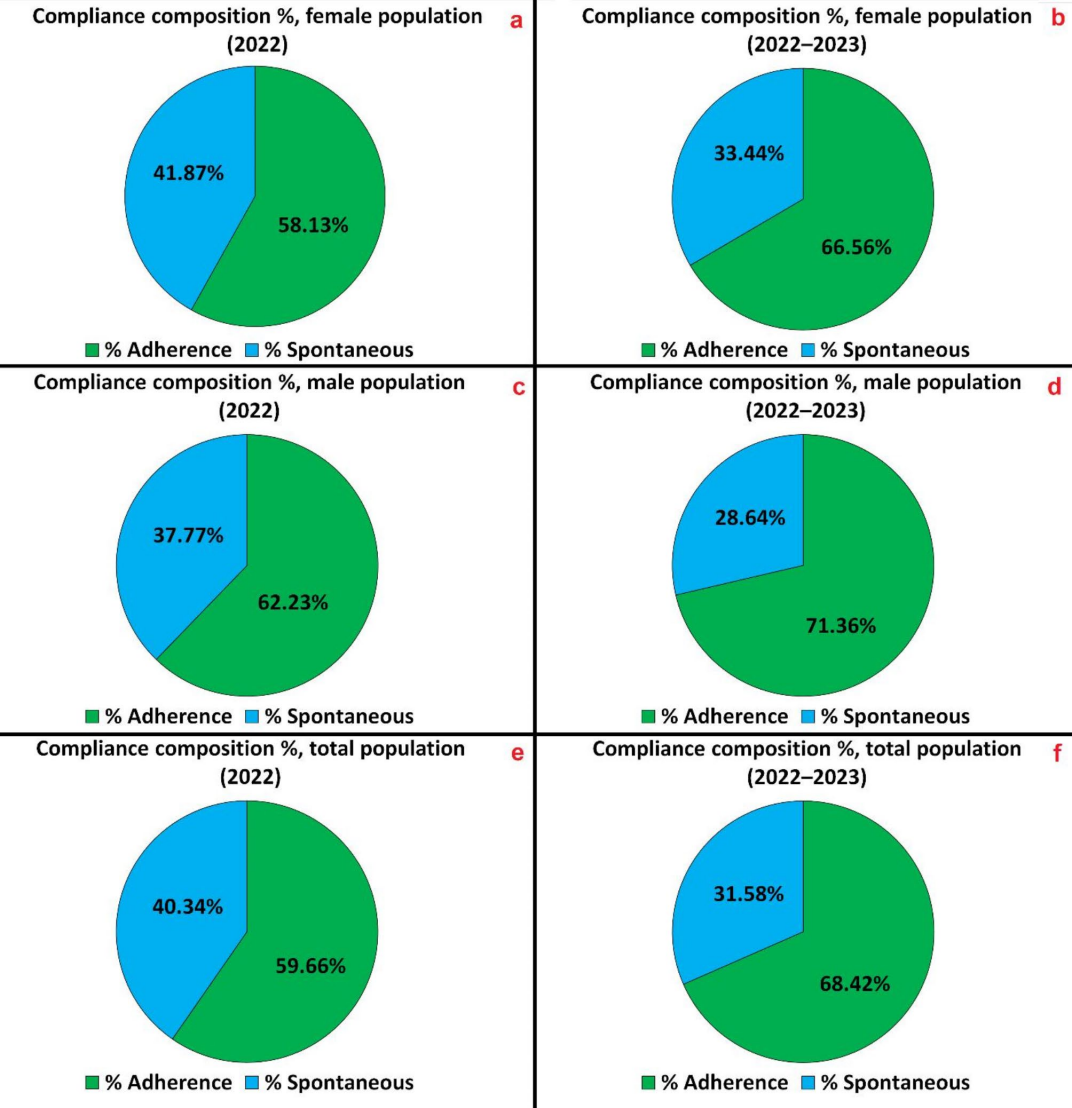
HCV screening compliance, with informed and spontaneous adherences evolution between 2022 and 2022–2023, for the Ferrara’s province: Female: (**a**) 2022. (**b**) 2022–2023. Male: (**c**) 2022. (**d**) 2022–2023. Total: (**e**) 2022. (**f**) 2022–2023.

Compliance distribution between informed and spontaneous adherence is shown in Fig. 4 (referred to Table S6 in Supplementary Material). In this figure, six pie charts display the participants’ compliance to the screening, categorized dependently on their afference to “Adherence” and “Spontaneous” groups (as presented in the “Protocol and Enlisting” subchapter), and sorting them in different panels by time frames (2022: a, c,e – 2023: b, d,f) and biological sexes (female subgroup: a,b – male subgroup: c,d – total: e,f).

Data showed as per TARGET 1 population it was observed that in the 2022 year, the 47.11% of the invited female population adhered to the 1st level of screening, while only the 27.66% of the invited male population attended; in the 2022–2023 time frame, the compliance from the two groups increased respectively to 64.09% and 40.11%. Subsequently, 0.97% male adherents were diagnosed positive to the 1st level of screening against 0.55% female adherents in 2022, with a total average of 0.71% positive cases on the total number of adherents (247 positives over 34,998 adherents), while in the 2022–2023 time frame 1.12% male adherents were diagnosed positive against 0.55% female adherents, total average of 0.77% positive cases (393 positives over 50,768 adherents).

In the 2022–2023 time frame, the TARGET 1 population (born between 1969 and 1989) registered a positivity rate to the 1st level of screening of 0.71%, while TARGET 2 population (SerD) showed a positivity rate of 12.38%, and TARGET 3 population (prison) displayed a 10.00% HCV antibodies positivity rate.

The distribution of the various genotypes in patients positive to the 2nd level of screening, in the 2022–2023 time frame, is: 1a (35.06%), 1b (27.27%), 3a (22.08%), 2a/2c (10.39%) and 4a/4c/4d (5.19%).

Regarding to positivity to the 2nd level of screening in the 2022–2023 time frame, Italian male population registered 50.65% of the positive cases, Foreign female population 18.18%, Foreign male population 15.58%, and Italian female population 15.58% as well.

The final detection rates for this HCV screening campaign, calculated between tested individuals and positive patients to the 2nd level of screening, are the following for 2022: TARGET 1 population: 1.14; TARGET 2 population: 0.00; TARGET 3 population: 34.15; TOTAL: 1.33. For what concerns the 2022–2023 period, the detection rates were: TARGET 1 population: 1.52; TARGET 2 population: 19.05; TARGET 3 population: 21.95; TOTAL population: 1.72.

For what concerns the screening compliance, the “Adherence” subgroup by patients informed from the national invitation protocol registered a 58.13% participation in 2022, and a 66.56% participation in the 2022–2023 period for the female population, while the male population registered a 62.23% participation in 2022 and a 71.36% in 2022–2023. Overall, the total citizenship compliance for the “Adherence” subgroup has been 59.66% in 2022 and 68.42% in the 2022–2023 period. For what concerns the “Spontaneous” requests for HCV testing during other routine blood samplings, it was observed a 41.87% participation in 2022 and a 33.44% in the 2022–2023 period for the female population, and a 37.77% participation in 2022 to 28.64% in the 2022–2023 period for the male population. Consequently, the overall citizenship compliance has been 40.34% in 2022 and 31.58% in the 2022–2023 period.

Finally, the total compliance registered to HCV screening of the three TARGET populations has been 37.41% in 2022 and 51.83% in the 2022–2023 time frame. In particular, TARGET 1 population had a screening compliance of 37.33% in 2022 and of 52.00% in the two years considered.

## Discussion

Various demographical trends have been observed from the data obtained. First of all, female patients looked more inclined being tested than male patients, while regarding test positivity, male patients are more commonly affected from HCV infection rather than females. As per Table 1 data, older participants to the screening showed higher compliance, as well as higher HCV antibodies (1st level) positivity, as expected from the preliminary studies that identified the critical segment of population selected as TARGET 1 for the screening^19^.

TARGET 2 population (SerD) showed the highest positivity rate between the three TARGET populations, followed closely from TARGET 3 population. This is expected from literature^2^, as hepatitis C transmission and chronic degeneration happens (being HCV a bloodborne virus) more frequently from drugs injection transmission with re-used syringes, followed by non-disposable surgical devices reuse.

In respect to the genotype subtypes mapping, genotypes 1a, 1b and 3a are the most common as far as shown from the data in the Ferrara’s province. In particular, genotype 1b proved to be the more common between female population, especially on Foreign patients. *A*ll HCV genotype 4-type infections were on the local male patients of Italian origin, in contrast with the common association of these specific subtypes with infections from Africa and Middle East regions^12–14,30,31^.

Italian male population represent the majority (half) of HCV positive cases to the 2nd level of screening, with the other three subgroups (Foreign males, Italian females, and Foreign females) more or less evenly dividing the remaining half of the positive cases, with a slight edge for the Foreign female population. This magnifies what already known in literature^2^ for the Italian subgroup, as the European male-to-female ratio was reported as of 2.2:1, while in Ferrara’s case we have 3.3:1 for the local native population. If we consider the Foreign population subgroup, instead, as per actual data the statistic plunges, with a male-to-female ratio of infection of 0.9:1. Overall, on the total population of positive cases displays a male to female positivity ratio of 2.0:1, nearing the European values.

The overall Foreign population, despite being 10.51% of the total local population (35.659 of 339.287 people, 2023 census data^32^) represents 31.17% of the HCV positive patients, showing an higher HCV infection incidence compared to the Italian population.

The invitation protocol proposed greatly improved the screening compliance, by comparing the two periods of time, for a net total of 50.768 people tested over TARGET 1 population, and 51.283 between the three TARGET populations.

Regarding the screening compliance, the “Adherence” subgroup showed an increase in the participation from 2022 to 2022–2023 period for both the female and male population. Consequently, the “Spontaneous” subgroup participation decreased in the same time frames, since most of these adherents already underwent screening in the first year, while remaining still a huge portion of the total compliance (31.58%), highlighting the efficiency of the innovative medical protocol proposed.

Finally, the total compliance to HCV screening of the three TARGET populations increased of + 14.42% between 2022 and 2023. If we consider the most numerous TARGET population, TARGET 1, the screening compliance increased of + 14.67%.

As per observations on data, the highest Italian participants’ compliance for HCV screening between the 2022 start and 2023 end was obtained in Ferrara’s province (51.83% overall, while for TARGET 1 population we registered 52.0% of adherents in respect to the national data of 21.0%^33^), thanks to the double invitation protocol implemented from the team. Furthermore, a reliable HCV genotypes and subtypes characterization has been drafted.

For what concerns the therapeutical treatment, the DDA’s of choice (see Direct-Acting Antiviral) proved to have high specificity in respect to the virus, and patients showed high compliance to their usage.

Overall, the protocol sensibilized and responsibilized both patients and healthcare professionals, thanks to the informative policy clarity and transparency, and the preparation beforehand of the tags and vials needed for the venous blood sampling.

Therefore, the double invitation approach greatly improved both groups’ compliances, allying doubts on the participation and hastening the sampling and screening process.

Even so, it remained difficult to motivate and endorse people to the therapeutic course after verified positivity, due to the general attitude on the knowledge of being ill, as described in the “Criticalities” subchapter. As per literature [33], on a national scale, TARGET 1 population invited and adherent to the HCV screening are respectively the 31.3% and 21.0% of the eligible citizens, TARGET 2 population the 49.5% and 59.9% of the Addiction Service patients, TARGET 3 population the 69.0% and 85.9% of the inmates. For this reason, further work and funds, together with new protocols to solicit treatment adherence between the population, are required. A possible action could be gender-customized medical pathways, in order to follow different sensibilities in the pathological matter, thus enhancing this last step of the HCV prevention and eradication program.

## Electronic supplementary material

Below is the link to the electronic supplementary material.

Supplementary Material 1

## Data Availability

Data is provided within the manuscript and supplementary information files.

## Abbreviations

Ab: Antibodies
AUSL: Aziende Unità Sanitarie Locali – Local Health Authority
CLIA: ChemiLuminescence ImmunoAssay
DAAs: Direct-Acting Antivirals
HCV: Hepatitis C
IgG: Immunoglobulin *G*
LIA: Line ImmunoAssay
NGS: Next-Generation Sequencing Technologies
PCR: Polymerase Chain Reaction
PLC: Primary Liver Cancer
RT-qPCR: Reverse Transcription quantitative Polymerase Chain Reaction
SerD: Servizio per le Dipendenze – Addiction Service
STP: Stranieri Temporaneamente Presenti – Foreigners temporarily present
UOC: Unità Operativa Complessa – Complex Operative Unit

## References

[1] Hepatitis C. World Health Organization, Accessed 13 November, 2024; https://www.who.int/news-room/fact-sheets/detail/hepatitis-c. Published 9 April, 2024.

[2] Hepatitis C Annual Epidemiological Report for 2020 surveillance report, ECDC European Centre for Diseases prevention and Control.

[3] Kondili, L. A. et al. Prevalence of hepatitis C virus estimates of undiagnosed individuals in different Italian regions: A mathematical modelling approach by route of transmission and fibrosis progression with results up to January 2021. *New Microbiol.* **45**(4), 249–259 (2022). 36066213

[4] Soza, A., Riquelme, A. & Arrese, M. Routes of transmission of hepatitis C virus. *Ann. Hepatol.* **9**(1), S30–S33 (2010). 20713992

[5] Society for Maternal-Fetal Medicine (SMFM). Electronic address: pubs@smfm.org; Hughes, B.L., Page, C.M. & Kuller, J.A. Hepatitis C in pregnancy: Screening, treatment, and management. *Am. J. Obstet. Gynecol.* 217(5), B2–B12 (2017). 10.1016/j.ajog.2017.07.039 28782502

[6] Cirrhosis of the Liver. Cleveland Clinic, accessed 13 November 2024; https://my.clevelandclinic.org/health/diseases/15572-cirrhosis-of-the-liver/. Published 26 July 2023.

[7] Fiehn, F., Beisel, C. & Binder, M. Hepatitis C virus and hepatocellular carcinoma: Carcinogenesis in the era of direct-acting antivirals. *Curr. Opin. Virol.* **67**, 101423 (2024). 38925094 10.1016/j.coviro.2024.101423

[8] Le terapie per curare l’epatite C. EPAC, accessed 13 November, 2024; https://www.epac.it/patologie/nuove-terapie/.

[9] Malattie Infettive – Epatite C – Terapia. Ministero Della Salute, accessed November 13, 2024; https://www.salute.gov.it/portale/malattieInfettive/dettaglioSchedeMalattieInfettive.jsp?lingua=italiano&id=118&area=Malattie%20infettive&menu=indiceAZ&tab=6/. Published 27 July, 2023.

[10] Cos’è Sofosbuvir/Velpatasvir. EPAC, accessed 13 November, 2024; https://www.epatitec.info/terapie/terapia-sofosbuvir-velpatasvir/.

[11] Huff, J. & Andersen, R. Glecaprevir/Pibrentasvir: The first 8-week, pangenotypic HCV treatment regimen for patients 12 years of age and older. *Ann. Pharmacother.* **54**(3), 262–276 (2020). 31537106 10.1177/1060028019877128

[12] Table 1 - Confirmed HCV genotypes/subtypes. ICTV, accessed 13 November, 2024; https://ictv.global/sg_wiki/flaviviridae/hepacivirus/table1/. Published March, 2023.

[13] Messina, J. P. et al. Global distribution and prevalence of hepatitis C virus genotypes. *Hepatology* **61**, 77–87 (2015). 25069599 10.1002/hep.27259 PMC4303918

[14] Gower, E. et al. Global epidemiology and genotype distribution of the hepatitis C virus infection. *J. Hepatol.* **61**(1), S45–S57 (2014). 25086286 10.1016/j.jhep.2014.07.027

[15] Zago, D. et al. Circulating genotypes of hepatitis C virus in italian patients before and after the application of wider access criteria to HCV treatment. *Open Microbiol. J.*10.2174/18742858-v16-e2205300 (2022).

[16] Ansaldi, F. et al. Different seroprevalence and molecular epidemiology patterns of hepatitis C virus infection in Italy. *J. Med. Virol.* **76**(3), 327–332 (2005). 15902713 10.1002/jmv.20376

[17] Epatite C, al via in Emilia-Romagna lo screening gratuito. Regione Emilia Romagna - Salute. Epatite C, al via in Emilia-Romagna lo screening gratuito/, accessed 13 Nov, 2024. Published January 25, 2022.

[18] Epatite C, la Regione conferma anche per il 2023 lo screening gratuito per i nati dal 1969 al 1989. Regione Emilia Romagna – Salute, Epatite C, la Regione conferma anche per il 2023 lo screening gratuito per i nati dal 1969 al 1989/, accessed 13 November, 2024. Published 14 February, 2023.

[19] Quotidianosanità.it – Studi e Analisi - Epatite C. In Italia ci sono ancora 380mila sieropositivi. Ecco perché dobbiamo accelerare lo screening, accessed 13 November, 2024; https://www.quotidianosanita.it/studi-e-analisi/articolo.php?articolo_id=100132. Published 18 November, 2021.

[20] DECRETO 14/05/2021, “Esecuzione dello screening nazionale per l’eliminazione del virus dell’HCV.” (21A04075) (GU Serie Generale n.162 del 08-07-2021), accessed 13 November, 2024. https://www.gazzettaufficiale.it/eli/id/2021/07/08/21A04075/sg/.

[21] Viral Hepatitis and Liver Disease - Laboratory Tests - Hepatitis C. U.S. Department of Veteran Affairs, accessed 13 November, 2024. https://www.hepatitis.va.gov/hcv/screening-diagnosis/laboratory-tests.asp/.

[22] Yaari, A. et al. Detection of HCV salivary antibodies by a simple and rapid test. *J. Virol. Methods* **133**(1), 1–5 (2006). 16360219 10.1016/j.jviromet.2005.09.009

[23] de Medina, M. et al. Detection of anti-hepatitis C virus antibodies in patients undergoing dialysis by utilizing a hepatitis C virus 3.0 assay: Correlation with hepatitis C virus RNA. *J. Lab. Clin. Med.* **132**(1), 73–75 (1998). 9665375 10.1016/s0022-2143(98)90028-2

[24] Sonal Kumar, M. D. Epatite, C, Cronica. M. S. D. Accessed 13 November, 2024; https://www.msdmanuals.com/it-it/professionale/malattie-del-fegato-e-delle-vie-biliari/epatite/epatite-c-cronica/. Published August, 2022.

[25] Screening dell’infezione da HCV in Emilia-Romagna. Regione Emilia Romagna – Salute, Accessed 13 November, 2024; https://salute.regione.emilia-romagna.it/notizie/regione/2023/novembre/epatite_c_tabella_riepilogativa_i_semestre_2023.pdf/. Published August, 2022.

[26] Barnett, N. W. & Francis, P. S. CHEMILUMINESCENCE|Overview. Editor(s): Worsfold P, Townshend A, Poole C. *Encyclopedia of Analytical Science* (Second Edition), Elsevier, 506–511 (2005).

[27] Jalali, M., Zaborowska, J. & Jalali, M. Chapter 1 - The Polymerase Chain Reaction: PCR, qPCR, and RT-PCR, Editor(s): Jalali M, Saldanha FYL, Jalali M. *Basic Science Methods for Clinical Researchers*, Academic Press, 1–18 (2017).

[28] Gallagher, S. & Chakavarti, D. Immunoblot analysis. *J. Vis. Exp.* **20**(16), 759 (2008). 10.3791/759 PMC2583035 19066547

[29] Satam, H. et al. Next-generation sequencing technology: Current trends and advancements. *Biology* **12**(7), 997 (2023). 37508427 10.3390/biology12070997 PMC10376292

[30] Asselah, T. & Bourlière, M. Hepatitis C virus: Current and evolving treatments for genotype 4. *Gastroenterol. Clin. North Am.* **44**(4), 859–870 (2015). 26600224 10.1016/j.gtc.2015.07.013

[31] Kamal, S. M. & Nasser, I. A. Hepatitis C genotype 4: What we know and what we don’t yet know. *Hepatology* **47**(4), 1371–1383 (2008). 18240152 10.1002/hep.22127

[32] Cittadini stranieri 2023 - provincia di Ferrara. TuttItalia, accessed 13 November, 2024; https://www.tuttitalia.it/emilia-romagna/provincia-di-ferrara/statistiche/cittadini-stranieri-2023/. Published 1 January, 2023.

[33] Screening nazionale HCV - Regioni: al via i piani regionali. abbviePRO, accessed 13 November, 2024; https://www.abbviepro.com/it/it/virology/screening-nazionale-hcv/legge-e-delibere-regionali.html. Published 1 January, 2023.

