# Supplementary material for: Italian screening protocol and genotypes characterization for HCV elimination (2022–2023) in Ferrara’s province: a real-world study
Source: Sci Rep. 2025 Mar 8;15:8087. doi: 10.1038/s41598-025-92654-w (PMC11890607; doi:10.1038/s41598-025-92654-w)
Supplement: Supplementary file 1 — Supplementary Material 1 [file 41598_2025_92654_MOESM1_ESM.pdf]

Supplementary Material

Genotype	Female	Male	Total	Genotype percentage of						
				female on total female population	male on total male population	female on total genotype population	male on total genotype population	female on total positive population	male on total positive population	total on total positive population
1a	7	20	27	26.92%	39.22%	25.93%	74.07%	9.09%	25.97%	35.06%
1b	10	11	21	38.46%	21.57%	47.62%	52.38%	12.99%	14.29%	27.27%
2a/2c	4	4	8	15.38%	7.84%	50.00%	50.00%	5.19%	5.19%	10.39%
3a	5	12	17	19.23%	23.53%	29.41%	70.59%	6.49%	15.58%	22.08%
4a/4c/4d	0	4	4	0.00%	7.84%	0.00%	100.00%	0.00%	5.19%	5.19%
Total	26	51	77	33.77%	66.23%	33.77%	66.23%	33.77%	66.23%	100.00%

Table S1: *HCV genotype percentage profiling per biological sexes and relative percentage ratios.*

	Genotype (No. HCV positives)										
Sex (Origin)	1a (27)	1b (21)	2a/2c (8)	3a (17)	4a/4c/4d (4)	Total (77)	% 1a (27)	% 1b (21)	% 2a/2c (8)	% 3a (17)	% 4a/4c/4d (4)	% genotype total (77)
Female (IT)	6	2	3	3	0	14	42.86%	14.29%	21.43%	21.43%	0.00%	18.18%
Female (FO)	1	8	1	2	0	12	8.33%	66.67%	8.33%	16.67%	0.00%	15.58%
Male (IT)	19	5	4	7	4	39	48.72%	12.82%	10.26%	17.95%	10.26%	50.65%
Male (FO)	1	6	0	5	0	12	8.33%	50.00%	0.00%	41.67%	0.00%	15.58%
Foreign	2	14	1	7	0	24	8.33%	58.33%	4.17%	29.17%	0.00%	31.17%
Italy	25	7	7	10	4	53	47.17%	13.21%	13.21%	18.87%	7.55%	68.83%

Table S2: *HCV genotypes profiling percentage per origin and biological sex over female (26), male (51), foreign (24), Italian (53) and total (77) positive population.*

	Genotype (No. HCV positives)										
Origin	1a (27)	1b (21)	2a/2c (8)	3a (17)	4a/4c/4d (4)	Total (77)	% 1a (27)	% 1b (21)	% 2a/2c (8)	% 3a (17)	% 4a/4c/4d (4)
Foreign	2	14	1	7	0	24	7.41%	66.67%	12.50%	41.18%	0.00%
Italy	25	7	7	10	4	53	92.59%	33.33%	87.50%	58.82%	100.00%
Total	27	21	8	17	4	77					

Table S3: *HCV genotypes profiling percentage per origin over single genotype populations.*

Positive Patients per Years of Birth					
Years of birth	Female	Male	Foreign	Italy	Total
1964	0	1	0	1	1
1965	0	0	0	0	0
1966	0	0	0	0	0
1967	0	1	0	1	1
1968	0	0	0	0	0
1969	2	4	0	6	6
1970	1	4	1	4	5
1971	2	3	1	4	5
1972	3	5	2	6	8
1973	3	4	1	6	7

1974	3	0	1	2	3
1975	3	0	2	1	3
1976	2	6	4	4	8
1977	1	3	1	3	4
1978	0	5	1	4	5
1979	2	3	0	5	5
1980	0	2	0	2	2
1981	0	2	1	1	2
1982	0	3	3	0	3
1983	0	0	0	0	0
1984	2	2	4	0	4
1985	0	1	0	1	1
1986	0	0	0	0	0
1987	1	0	0	1	1
1988	0	1	1	0	1
1989	0	0	0	0	0
1990	1	0	1	0	1
1991	0	0	0	0	0
1992	0	0	0	0	0
1993	0	1	0	1	1
Total	26	51	24	53	77

Table S4: *Profiling of positive cases depending on years of birth over origin and biological sex.*

**Positive patients genotypes overview**		
Sex (Origin)	Years of birth	HCV genotype
Female (Italy)	1969	1a
	1969	3a
	1971	1b
	1972	3a
	1973	1a
	1973	2a/2c
	1974	2a/2c
	1974	3a
	1975	2a/2c
	1976	1a
	1977	1a
	1979	1a
	1979	1b
	1987	1a
Female (Foreign)	1974	2a/2c
	1971	1b
	1973	1b
	1984	1a
	1984	1b
	1975	1b
	1970	1b

	1972	1b
	1972	1b
	1975	1b
	1976	3a
	1990	3a
Male (Italy)	1964	1a
	1967	3a
	1969	1a
	1969	1a
	1969	1b
	1969	2a/2c
	1970	1a
	1970	1b
	1970	1b
	1970	1b
	1971	1a
	1971	2a/2c
	1971	3a
	1972	1a
	1972	1a
	1972	1a
	1972	3a
	1972	4a/4c/4d
	1973	1a
	1973	1a
	1973	1b
	1973	4a/4c/4d
	1976	1a
	1976	1a
	1976	4a/4c/4d
	1977	1a
	1977	2a/2c
	1978	1a
	1978	3a
	1978	3a
	1978	3a
	1979	1a
	1979	3a
	1979	4a/4c/4d
	1980	1a
	1980	2a/2c
	1981	1a
	1985	1a
	1993	1a
Male (Foreign)	1977	1a

	1976	1b
	1984	1b
	1982	1b
	1982	1b
	1984	1b
	1978	3a
	1988	1b
	1976	3a
	1976	3a
	1981	3a
	1982	3a

Table S5: *Profiling of HCV genotypes per year, biological sex and origin.*

	**COMPLIANCE**			
	**2022**		**2022–2023**	
	Adherence	Spontaneous	Adherence	Spontaneous
Female	58.13%	41.87%	66.56%	33.44%
Male	62.23%	37.77%	71.36%	28.64%
Total	59.66%	40.34%	68.42%	31.58%

Table S6: *Compliance percentage contributes: informed adherence and spontaneous adherence percentage profiling over patients tested per biological sex.*
